# Clinical Characteristics, Patterns of Care, and Treatment Outcomes of Radiation-Associated Sarcomas

**DOI:** 10.3390/cancers16101918

**Published:** 2024-05-18

**Authors:** Rohit Raj, Han Gil Kim, Menglin Xu, Tyler Roach, David Liebner, David Konieczkowski, Gabriel Tinoco

**Affiliations:** 1 Department of Radiation Oncology, The Ohio State University Comprehensive Cancer Center, Columbus, OH 43210, USA; rohit.raj@osumc.edu (R.R.); david.konieczkowski@osumc.edu (D.K.); 2 Department of Medical Oncology, The Ohio State University Comprehensive Cancer Center, Columbus, OH 43210, USA; hangil.kim@osumc.edu (H.G.K.); menglin.xu@osumc.edu (M.X.); david.liebner@osumc.edu (D.L.); 3 Department of Internal Medicine, The Ohio State University Wexner Medical Center, Columbus, OH 43210, USA; tyler.roach@osumc.edu

**Keywords:** radiation therapy, radiation-associated, sarcoma, secondary malignancy, soft tissue

## Abstract

**Simple Summary:**

Radiation-associated sarcomas pose a significant clinical challenge due to their complexity, aggressiveness, and poor prognosis. Our retrospective study is one of the largest descriptions of radiation-associated sarcomas, aiming to shed light on their clinicopathologic characteristics and highlight treatment approaches. Our research found a significant association between high radiation-associated sarcoma histologic grade and inferior clinical outcomes. We also found that localized radiation-associated sarcoma patients who underwent margin-negative resections lived significantly longer than their counterparts. Our analysis thus confirms the paramount role of tumor grade and the central importance of margin-negative oncologic resection in determining clinical outcomes for radiation-associated sarcomas.

**Abstract:**

Radiation-associated sarcomas (RASs) are rare tumors with limited contemporary data to inform prognostication and management. We sought to identify the clinical presentation, patterns of care, and prognostic factors of RASs. RAS patients treated at a single institution from 2015 to 2021 were retrospectively reviewed for clinicopathologic variables, treatment strategies, and outcomes. Thirty-eight patients were identified with a median follow-up of 30.5 months. The median age at RAS diagnosis was 68.4 years (27.9–85.4), with a median latency from index radiotherapy (RT) of 9.1 years (3.7–46.3). RAS histologies included angiosarcoma (26%), undifferentiated pleomorphic sarcoma (21%), and osteosarcoma (18%). Most were high-grade (76%). Genomic profiling revealed low tumor mutational burden, frequent inactivating TP53 mutations (44%), CDKN2A deletions (26%), and MYC amplifications (22%), particularly in breast angiosarcomas. Of 38 patients, 33 presented with localized disease, 26 of whom were treated with curative intent. Overall, the median progression-free survival (PFS) was 9.5 months (1.4–34.7), and the overall survival (OS) was 11.1 months (0.6–31.6). Patients with localized vs. metastatic RASs had a longer PFS (HR, 3.0 [1.1–8.5]; *p* = 0.03) and OS (HR, 3.0 [1.04–8.68]; *p* = 0.03). Among localized RAS patients, high grade was associated with shorter OS (HR, 4.6 [1.04–20.30]; *p* = 0.03) and resection with longer OS (mean 58.8 vs. 6.1 months, HR, 0.1 [0.03–0.28]; *p* < 0.001). Among patients undergoing resection, negative margins were associated with improved OS (mean 71.0 vs. 15.5 months, HR, 5.1 [1.4–18.2]; *p* = 0.006). Patients with localized disease, particularly those undergoing R0 resection, demonstrated significantly better outcomes. Novel strategies are urgently needed to improve treatment outcomes in this challenging group of diseases.

## 1. Introduction

More than half of all cancer patients will receive radiotherapy (RT) as part of their oncologic treatment [1]. Although RT is oncologically highly effective, it is associated with significant morbidity in a subset of patients, including the development of secondary malignancies. Radiation-associated sarcomas (RASs) are rare, occurring in <1% of patients who receive RT but accounting for up to 5% of all sarcomas [2,3,4,5,6,7]. As the use of RT increases along with post-treatment survival, due to improvements in overall treatment efficacy, there is a growing population of radiation-exposed cancer survivors at risk of RASs [8]. This is particularly concerning given the worse outcomes for RASs compared with sporadic sarcomas [3,4,5,9,10].

Although several studies have documented RAS outcomes in small cohorts, there are limited data regarding prognostic features for outcomes in RASs. An analysis of the presentation, treatment, and outcomes of RASs provides an opportunity to understand the implications of this diagnosis in the modern era of sarcoma management. Such an understanding may be further enhanced by an investigation for potentially actionable genomic abnormalities; however, there are currently limited data on the genomic landscape of RT-associated malignancies [11,12,13].

This study aimed to describe the clinical presentation and patterns of care of RAS patients seen at our institution; evaluate the clinical, histologic, and treatment features of these RAS patients and correlate these data with patient outcomes; and analyze next-generation sequencing (NGS) data collected from these cases to identify potentially actionable genomic abnormalities.

## 2. Materials and Methods

### 2.1. Identification of Patient Cohort

This institutional retrospective study was conducted with approval from The Ohio State University Institutional Review Board (2021C0013). Patients evaluated at The Ohio State University Comprehensive Cancer Center between 1 January 2015 and 20 January 2021 for the treatment of RASs were included. Pathology reports and medical records were reviewed to identify authentic RAS cases using criteria similar to those of Cahan et al.: (1) histologic confirmation of a sarcoma distinct from the patient’s prior malignancy; (2) tumor arising within, or adjacent to, a previously irradiated field; and (3) tumor occurring at least 6 months after the completion of index RT [3,14,15,16].

### 2.2. Clinical Demographics and Radiation History

The clinical variables collected included patient factors such as age at the time of RT for the index malignancy, age at RAS diagnosis, and sex. Disease factors such as index malignancy histology; RAS histology, grade, anatomic location, and size; and latency between RT for the index malignancy and the diagnosis of RAS were ascertained. Treatment factors such as surgery and margin status for RAS, systemic treatment, and repeated RT for RAS were identified.

### 2.3. Histopathologic and Molecular Analysis of RAS

RAS tumors were classified according to either the 2013 or 2020 WHO Classification of Tumours of Soft Tissue and Bone (depending on the date of RAS histologic diagnosis) and graded according to the French Federation Nationale des Centers de Lutte Contre le Cancer criteria. Tumor sizes were determined using surgical pathology reports, imaging studies, and clinical notes. NGS was performed on DNA extracted from formalin-fixed paraffin-embedded tissue of 27 cases using the commercially available Tempus platform.

### 2.4. Dosimetric Analysis of Index Radiation Dose Received at RAS Tumor Site

The DICOM plan for the patient’s index course of RT was available for 8 patients in the cohort. For these patients, the RAS gross tumor volume (GTV) was contoured using diagnostic imaging (CT, MR, and/or PET/CT) at the time of RAS diagnosis. This dataset was then registered to the index DICOM RT plan to determine index radiation doses for the RAS GTVs.

### 2.5. Outcome Data

Kaplan–Meier plots were used to evaluate locoregional control (LRC), progression-free survival (PFS), and overall survival (OS) in relevant subsets with comparisons via a log-rank test and a significance level of alpha = 0.05.

## 3. Results

**Figure 1:**
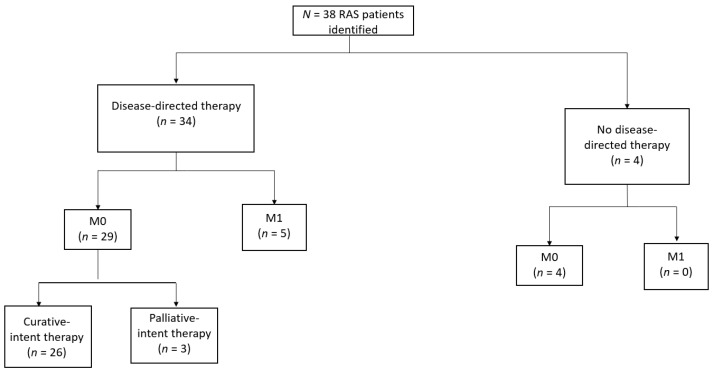
Study profile.

The review of pathology reports and medical records identified a cohort of 38 RAS patients (17 males and 21 females). Of these 38 RAS patients, 33 presented with localized disease, and 5 presented with metastatic disease (Figure 1).

The median age at RT for index malignancy was 55.3 years (range 0.2–77.5). Index malignancy histologies and treatments are detailed in Table 1. The most common index malignancies associated with a subsequent RAS were as follows: breast carcinoma (24%); prostate adenocarcinoma (21%); and head and neck squamous cell carcinoma and a distinct primary soft tissue sarcoma (13% of each histology). The radiation dose for index malignancy was known in 27 patients; among these, the median dose was 50.4 Gy (range 36.0–77.4). Among the eight localized RAS patients with available index radiotherapy DICOM plans, the median average dose for the RAS GTV was 36.2 Gy (range 0.6–49.9); the median maximum dose (D0.03cc) for the RAS GTV was 54.0 Gy (range 1.5–73.6).

Clinical and histologic features of RAS are provided in Table 2. The median latency following RT for index malignancies until RAS diagnosis was 9.1 years (range 3.7–46.3). The median age at RAS diagnosis was 68.4 years (range 27.9–85.4). The most common RAS histologies were as follows: angiosarcoma (26%); undifferentiated pleomorphic sarcoma (21%); and osteosarcoma (18%). The RAS tumor size was known for 34 (89%) patients, among whom the median size was 5.3 cm (range 0.4–30.5). Most RASs were high-grade (76%). RAS was most commonly located in the abdominopelvic region (40%), followed by the thorax and head and neck (each 26%). Genomic profiling was available in 71% of cases; the most common mutation was an inactivating *TP53* mutation (44%), followed by inactivating mutations in *BRCA2* and *RB1* (7% each) (Supplementary Table S1). The most common copy number variant was a copy number loss of *CDKN2A/B* (26% of cases), followed by *MYC* amplification/copy number gain (22% of cases); a total of 67% of the cases involving *MYC* amplification/copy number gain were radiation-associated angiosarcomas of the breast arising after treatment for breast carcinoma. The median tumor mutation burden (TMB) was 2.6 mutations/megabase (range 0.5–6.3).

Treatment modalities for RAS are depicted in Table 3. Of the 33 patients with localized RAS, 4 did not receive any disease-directed therapy owing to patient choice (n = 2), poor performance status (n = 1), or loss to follow-up (n = 1). Three were treated with palliative-intent systemic therapy alone, whereas the remainder (n = 26 [79%]) underwent curative-intent treatment including oncologic resection. Of the seven patients with localized RAS at presentation who did not undergo resection, the reasons for not undergoing surgery included patient choice (n = 2), unresectable disease (n = 2), inoperability due to medical comorbidities (n = 2), and simultaneous local and distant disease progression during neoadjuvant chemotherapy (n = 1). Among patients who underwent oncologic resection, surgical margins were microscopically negative (R0) in 73%, microscopically positive (R1) in 19%, and grossly positive (R2) in 8%.

Among the 26 patients with localized RAS treated with curative intent, 55% received systemic therapy, with the most common agents being doxorubicin (31%), paclitaxel (27%), and cisplatin and ifosfamide (15% received each agent). Systemic therapy was administered preoperatively, postoperatively, and both pre- and postoperatively in 41%, 24%, and 35% of patients who received systemic therapy as part of curative-intent management. Of the localized RAS patients treated with curative intent, 31% received re-irradiation. The median dose of re-irradiation was 50.4 Gy (range 45.0–57.5), with six patients (75%) receiving RT postoperatively and two patients (25%) receiving RT preoperatively. While the rationale for the administration of re-irradiation was not documented, the selection of postoperative re-irradiation was consistent with the general management paradigm of the most common histologic malignancy occurring in the anatomic region of the RAS (such as the common usage of postoperative radiotherapy in the management of head and neck squamous cell carcinomas) or following an R2 oncologic resection. Meanwhile, a salient feature of each case of preoperative re-irradiation was either a large RAS tumor or locoregionally extensive RAS disease. Due to the unavailability of particle therapy at our institution during the time period of the study, only one patient underwent postoperative re-irradiation with proton beam therapy (at an outside institution) as part of the curative-intent treatment of a periorbital RAS.

**Figure 2:**
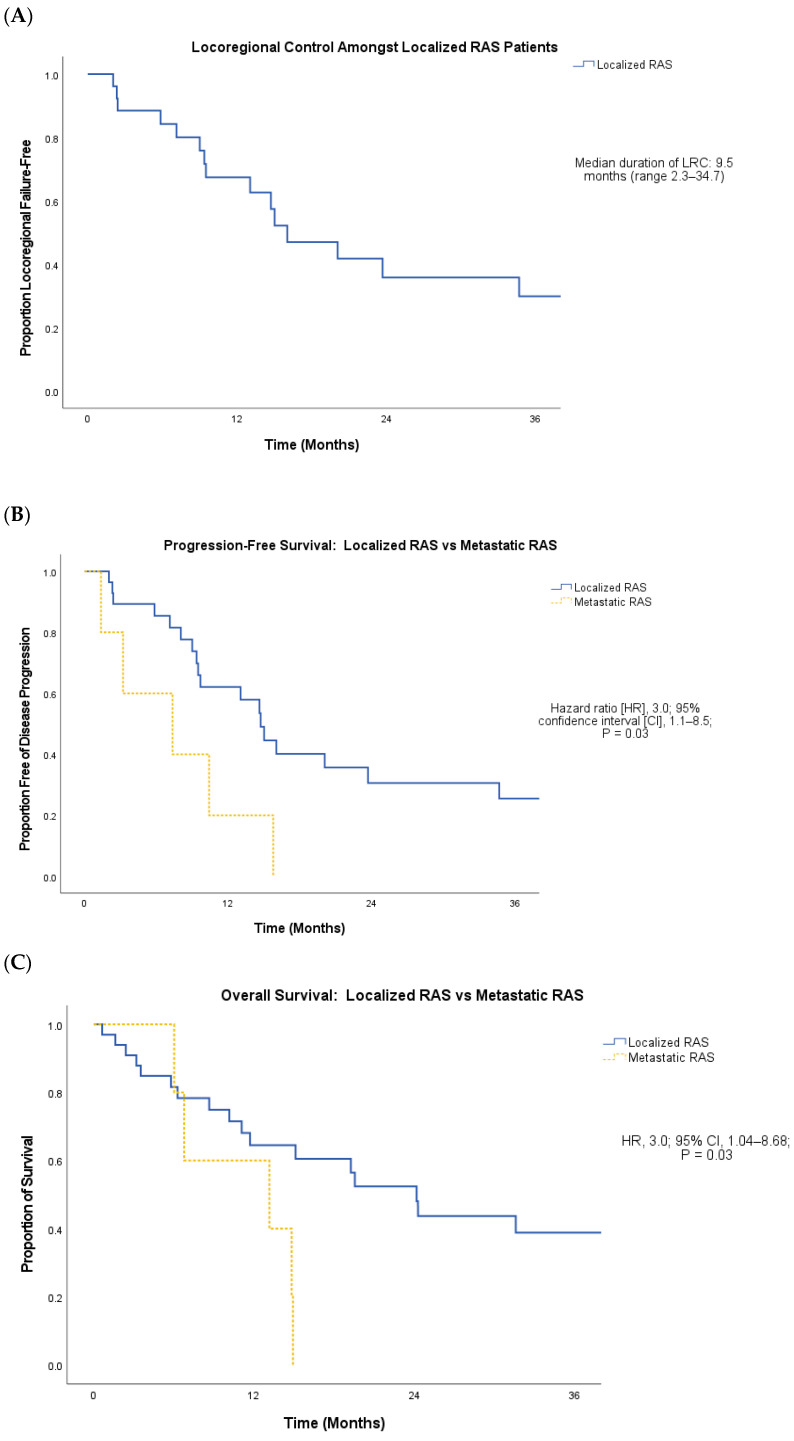
(**A**) Locoregional control (LRC) among localized radiation-associated sarcoma (RAS) patients. (**B**,**C**): Progression-free survival (PFS; (**B**)) and overall survival (OS; (**C**)) among localized RAS vs. metastatic RAS patients.

Among surviving patients, the median follow-up from the diagnosis of RAS was 30.5 months (range 3.7–98.5). Overall, the first site of failure was isolated local failure in 43%, isolated distant failure in 11%, and simultaneous local and distant failure in 29%. Four patients died before re-staging imaging and thus did not have failure location information. Among the entire cohort, the median progression-free survival (PFS) was 9.5 months (range 1.4–34.7), and the median overall survival (OS) was 11.1 months (range 0.6–31.6). Among patients who presented with localized RASs, the median duration of locoregional control (LRC) was 9.5 months (range 2.0–34.7; Figure 2A). Among the subset (n = 26/33) of localized RAS patients treated with curative intent, the median duration of LRC was 9.5 months (range 2.3–34.7); the median PFS was 9.6 months (range 2.3–34.7); and the median OS was 15.1 months (3.5–31.6).

**Figure 3:**
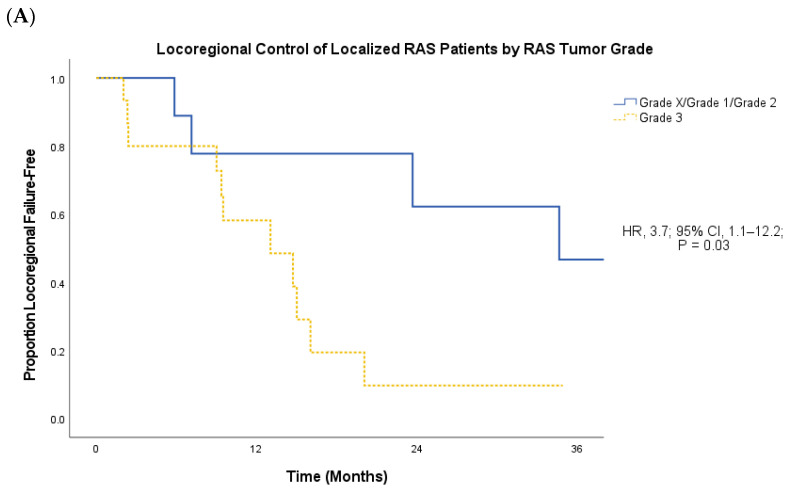

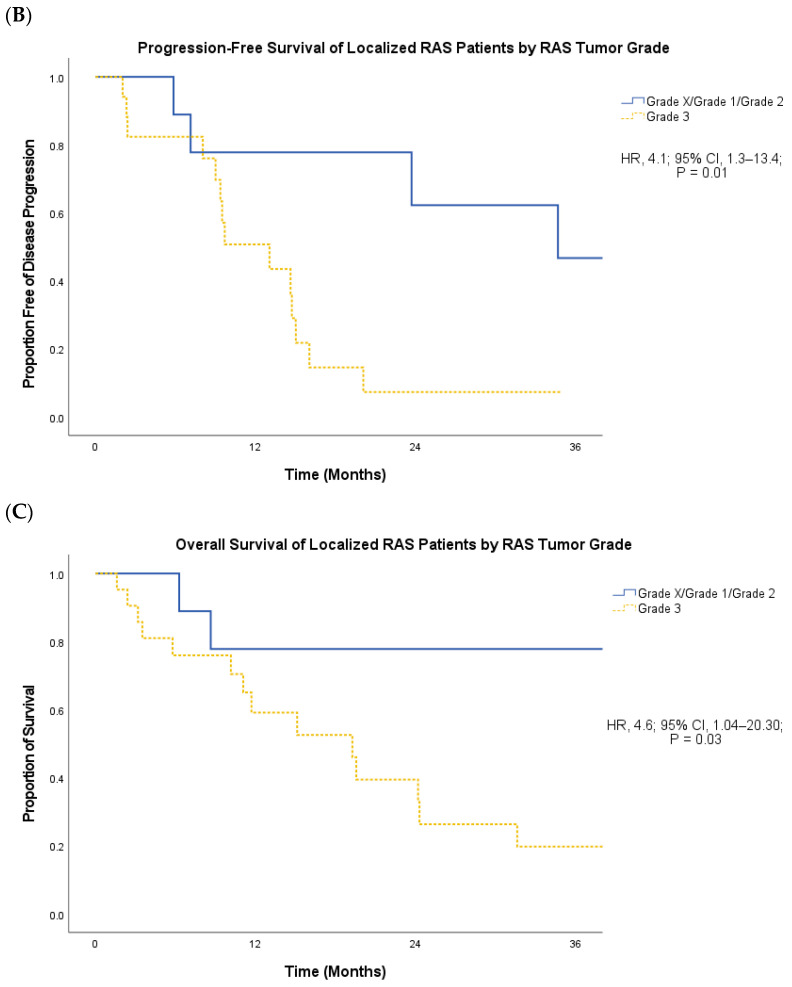
(**A**–**C**): LRC (**A**), PFS (**B**), and OS (**C**) of localized RAS patients based on RAS histologic grade.

Patients who presented with localized RAS had a significantly longer PFS (median 14.7 months vs. 7.4 months; hazard ratio [HR], 3.0; 95% confidence interval [CI], 1.1–8.5; *p* = 0.03; Figure 2B) and OS (median 24.2 months vs. 13.1 months; HR, 3.0; 95% CI, 1.04–8.68; *p* = 0.03; Figure 2C) compared to patients who presented with metastatic RAS. Among localized RAS patients, high RAS histologic grade was associated with significantly shorter LRC compared to low- and intermediate-grade disease (mean 13.5 months vs. 41.1 months; HR, 3.7; 95% CI, 1.1–12.2; *p* = 0.03; Figure 3A), as well as significantly shorter PFS (mean 12.6 vs. 41.1 months; HR, 4.1, 95% CI, 1.3–13.4; *p* = 0.01; Figure 3B) and OS (mean 20.7 vs. 79.4 months; HR, 4.6; 95% CI, 1.04–20.30; *p* = 0.03; Figure 3C).

**Figure 4:**
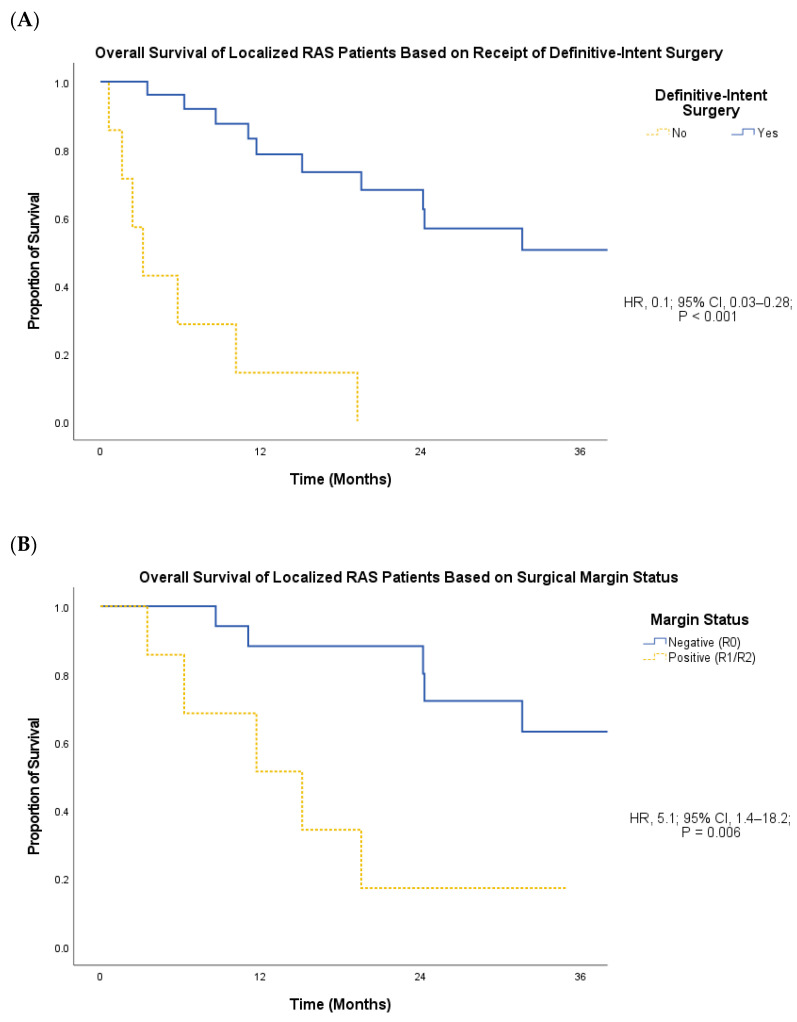
(**A**) OS of localized RAS patients based on receipt of definitive-intent oncologic resection. (**B**) OS of localized RAS patients treated with definitive-intent surgery based on surgical margin status.

The significant differences in PFS and OS by RAS histologic grade persisted when the metastatic RAS patients were included in the analysis, with a median PFS of 9.7 months for high-grade RAS vs. 34.7 months for low-/intermediate-grade RAS (HR, 3.8; 95% CI, 1.3–10.7; *p* = 0.008) and a mean OS of 18.6 months for high-grade RAS vs. 71.7 months for low-/intermediate-grade RAS (HR, 3.8; 95% CI, 1.1–13.0; *p* = 0.02). Localized RAS patients who underwent resection had a significantly longer OS compared to unresected localized RAS patients (mean 58.8 vs. 6.1 months; HR, 0.1; 95% CI, 0.03–0.28; *p* < 0.001; Figure 4A), with a 1-year OS of 65% versus 14%. There were insufficient local or systemic progression events to compare LRC or PFS by resection status. Nine of the eleven patients (82%) who experienced recurrence following R0 resection recurred locoregionally, while the remaining two patients (18%) recurred distally. In contrast, four of the six patients (67%) who experienced disease progression following an R1 or R2 resection progressed distally, while the remaining two patients (33%) progressed locoregionally. Among localized RAS patients who underwent resection, surgical margin status (R0 vs. R1/R2) was not associated with any significant differences in LRC (HR, 2.5; 95% CI, 0.8–8.4; *p* = 0.1) or PFS (HR, 2.4; 95% CI, 0.8–7.0; *p* = 0.1). However, R0 margin status was associated with significantly improved OS in comparison to R1/R2 margin status (mean 71.0 vs. 15.5 months; HR, 5.1; 95% CI, 1.4–18.2; *p* = 0.006; Figure 4B).

Finally, among patients managed with curative intent, there was no significant association between the receipt of systemic therapy and the duration of either PFS (HR, 1.6; 95% CI, 0.5–4.9; *p* = 0.4) or OS (HR, 0.6; 95% CI, 0.2–1.7; *p* = 0.4) nor was there any significant association between the receipt of re-irradiation and the duration of LRC (HR, 0.7; 95% CI, 0.1–3.0; *p* = 0.6), PFS (HR, 1.3; 95% CI, 0.4–3.8; *p* = 0.2), or OS (HR, 0.4; 95% CI, 0.1–1.9; *p* = 0.2).

## 4. Discussion

This study analyzes the clinical, histologic, and treatment characteristics and outcomes for a cohort of patients with both localized and metastatic RASs for whom contemporary diagnostic and treatment techniques were employed. The prognosis of RASs has generally been considered poor [4,17,18]; our data confirm this, with even localized RAS patients having a median survival of less than 12 months.

The drivers of outcome for RASs in this study were localized RAS at presentation, histologic grade, and, among localized RAS cases, the achievement of margin-negative resection. In our study, the median survival of localized low-/intermediate-/unassessed grade RAS was approximately double that of high-grade RASs. Our findings of inferior survival in high-grade disease are consistent with prior reports in RASs [19,20] as well as sporadic sarcomas [21]. Similarly, survival differences based on surgery and margin status were striking, with further improvements in the subgroup achieving R0 margins. This finding is consistent with prior results [3,4,5].

The major limitation of our study is the small size of our cohort, which limits the granularity of potential subset analyses, and its retrospective nature, which potentially limits generalizability. Within these limitations, clinical and pathologic data were quite complete in our cohort, although a minority of patients did not have outside RAS pathology available for review at the treating institution (5%, n = 2) or had incomplete data for some variables.

NGS data from our cohort identified relatively few actionable mutations. Inactivating *TP53* mutations were detected in nearly half of cases; this finding is consistent with prior data suggesting that inactivating *TP53* mutations occur in approximately one-third of RAS in patients without hereditary predisposition syndromes and that the inactivation of one *TP53* allele represents an early event in RAS tumor formation [22]. Additionally, *MYC* amplification was a recurrent finding in the breast angiosarcoma cases in our cohort; this finding is consistent with prior studies, which have found the consistent presence of high-level *MYC* amplification in radiation-associated angiosarcoma [23,24,25]. Moreover, *MYC* amplification may help genetically distinguish radiation-associated angiosarcomas from other RAS subtypes and from sporadic angiosarcomas, both of which demonstrate *MYC* amplification with substantially lesser frequency [19]. NGS data may have an impact on therapeutic strategies as well: for instance, one of six (17%) of the radiation-associated breast angiosarcomas in our cohort featured the amplification of the *KDR* gene encoding the amplification of the VEGFR2 tyrosine kinase receptor. An in vitro study demonstrated that 10% of patients with primary and secondary breast angiosarcomas exhibit activating *KDR* gene mutations [26]; based on their activity in vitro, VEGFR inhibitors could be studied in *KDR*-overexpressing radiation-associated breast angiosarcomas [27]. Meanwhile, another radiation-associated breast angiosarcoma in our cohort demonstrated a *PIK3CA* gain-of-function mutation; immunohistochemical studies in both radiation-associated and sporadic breast angiosarcomas have revealed the activation of the PIK3CA/Akt/mTOR pathway in a significant proportion of these tumors [28,29], making PIK3CA inhibitors a potentially relevant targeted therapy to explore. As the small sample size of single-institution studies of RASs limits the power to detect mutational signatures that may be common or unique to RAS histologies, multi-institutional or registry-based studies may provide the sample sizes required to identify clinically important genetic and transcriptomic features of RAS tumors.

Despite the potential for improvement in RAS outcomes with targeted therapies arising from genomic sequencing, the greatest future advances in RAS oncologic outcomes may arise from the addition of immunotherapy to RAS management paradigms. In a recent retrospective study, Hong and colleagues found that RAS tumors had a significantly higher mRNA level of PD-1 compared to sporadic sarcomas. In examining the oncologic outcomes of RAS patients treated with combination chemo-immunotherapy (doxorubicin-based chemotherapy with an anti-PD1 monoclonal antibody) versus doxorubicin-based chemotherapy alone, the authors reported that the patients treated with chemo-immunotherapy achieved a significantly higher objective response rate, longer OS, and longer PFS than those treated with chemotherapy alone [30]. Future investigations of the role of immunotherapy in RAS management should aim to include greater numbers of RAS patients (ideally through multi-institutional, international collaboration) and should be prospective in nature to further minimize the effect of confounding variables.

## 5. Conclusions

Ultimately, localized presentation, lower histologic grade, and margin-negative resection were associated with improved outcomes in our RAS cohort. Our findings support the central role of macroscopically complete, and ideally R0, surgical resection in the management of RAS. While our data do not directly inform the role of systemic therapies and re-irradiation in this rare context, the high rates of both local and distant progression and overall poor prognosis identified in this cohort highlight the opportunities for individualized, aggressive, multidisciplinary approaches to improve care for these challenging tumors.

## Figures and Tables

**Table 1:** Index malignancy disease and treatment characteristics.

Characteristics	Total (n = 38)
Median age at RT for index malignancy (range), years	55.3 (0.2–77.5)
Diagnosis, n (%)	
Breast carcinoma	9 (24)
Prostate adenocarcinoma	8 (21)
Head and neck squamous cell carcinoma	5 (13)
Sarcoma	5 (13)
Rectal adenocarcinoma	2 (5)
Lymphoma	2 (5)
Other	7 (18)
Treatment characteristics for primary malignancy	
Median radiation dose (range), Gy	50.4 (36.0–77.4)
Radiation field, n (%)	
Pelvis/groin	14 (37)
Head and neck	12 (32)
Breast	8 (21)
Trunk/chest wall	3 (8)
Spine	1 (3)
Upper extremity	1 (3)
Chemotherapy use, n (%)	21 (55)

RT = radiotherapy. Other: n = 1 of each of vulvar squamous cell carcinoma, cervical squamous cell carcinoma, retinoblastoma, adenoid cystic carcinoma, plasmacytoma, melanoma, and unknown histology.

**Table 2:** Radiation-associated sarcoma disease characteristics.

Characteristics	Total (n = 38)
Median age at RAS diagnosis (range), years Median latency after index RT (range), years	68.4 (27.9–85.4) 9.1 (3.7–46.3)
Location, n (%)	
Abdomen/pelvis	15 (40)
Head and neck	10 (26)
Thorax	10 (26)
Extremity	2 (5)
Other	1 (3)
Histology, n (%)	
Angiosarcoma	10 (26)
Undifferentiated pleomorphic sarcoma	8 (21)
Osteosarcoma	7 (18)
Leiomyosarcoma	4 (11)
Spindle cell sarcoma	3 (8)
Other undifferentiated sarcoma	2 (5)
Myxoinflammatory myofibroblastic sarcoma	1 (3)
Fibrosarcoma	1 (3)
Malignant biphasic neoplasm	1 (3)
Hemangiopericytoma	1 (3)
Histologic grade, n (%)	
Not assessed	5
Low	5 (15)
Intermediate	3 (9)
High	25 (76)
Next-generation sequencing data, n (%)	
*TP53* mutation (missense, nonsense, frameshift)	12 (44)
*CDKN2A/B* copy number loss	7 (26)
*MYC* amplification/copy number gain	6 (22)

RAS = radiation-associated sarcoma; RT = radiotherapy.

**Table 3:** Radiation-associated sarcoma treatment characteristics.

Localized RAS	
Surgery, n (%)	
No	7 (21)
Yes	26 (79)
Surgical margin status, n (%)	
Negative (R0)	19 (73)
Microscopic disease (R1)	5 (19)
Macroscopic disease (R2)	2 (8)
Chemotherapy, n (%)	18 (55)
Radiotherapy, n (%)	8 (24)
Median radiation dose, Gy (range; n = 8)	50.2 (44.0–57.5)
Metastatic RAS	
Palliative-intent surgery, n (%)	1 (20)
Chemotherapy, n (%)	
No	0 (0)
Yes	5 (100)
Palliative-intent radiotherapy, n (%)	3 (60)
Median radiation dose, Gy (range; n = 3)	39.0 (20.0–44.0)

## Data Availability

The data presented in this study are available on request from the corresponding author.

## Supplementary Materials

The following supporting information can be downloaded at https://www.mdpi.com/article/10.3390/cancers16101918/s1, Supplementary Table S1: Summary of next-generation sequencing results in radiation-associated sarcomas.
