# Supplementary material for: Clinical Characteristics, Patterns of Care, and Treatment Outcomes of Radiation-Associated Sarcomas
Source: Cancers (Basel). 2024 May 18;16(10):1918. doi: 10.3390/cancers16101918 (PMC11119080; doi:10.3390/cancers16101918)
Supplement: Supplementary file 1 [file cancers-16-01918-s001.zip › cancers-2969634-supplementary.pdf]

Supplemental Table S1. Summary of next-generation sequencing results in radiation-associated sarcomas (n = 27).

Category	Gene	Genetic alteration	[n, (%)]
Short nucleotide or amino acid variants			
	TP53	Missense, nonsense, and frameshift mutations	12 (44)
	BRCA2	G2274fs*17<br>L3045fs*17	2 (7)
	RB1	A106fs*4<br>p.L335 stop gain—LOF	2 (7)
	NF2	p.E347fs<br>F256fs*40	2 (5)
	NF1	p.D1067fs LOF	1 (4)
	PLCG1	p.R707Q splice region variant—GOF	1 (4)
	SMAD4	p.R361C missense LOF	1 (4)
	PIK3CA	p.H1047R missense GOF variant	1 (4)
	HRAS	p.G13V missense GOF	1 (4)
	MSH3	c.1653+1G>A splice region variant—LOF	1 (4)
	MUTYH	G382D	1 (4)
	DNMT3A	R635W—subclonal<br>R882H—subclonal	1 (4)
	SETD2	G1279*	1 (4)
	TERT	c.-57A>C variant—promoter mutation	1 (4)
	BAP1	L564fs*3	1 (4)
	ATRX	c.5698-2A>G splice region variant—LOF	1 (4)
	ZFHX3	p.E1900fs—LOF	1 (4)
	PAX5	p.A322fs—LOF	1 (4)
	ASXL1	K559fs*6	1 (4)
Copy number variants			
	CDKN2A/B	Copy number loss	7 (26)
	MYC	Amplification/copy number gain	6 (22)
	FGFR1	Amplification	2 (7)
	PDGFRA	Amplification/copy number gain	2 (7)
	RB1	Copy number loss	2 (7)
	PTEN	Copy number loss	2 (7)
	BIRC3	Amplification	1 (4)
	MDM2/CDK4	Amplification	1 (4)
	HGF	Amplification	1 (4)
	KDR	Amplification	1 (4)
	KIT	Amplification	1 (4)
	PDPK1	Copy number gain	1 (4)
	CCNE1	Copy number gain	1 (4)
	MCL1	Copy number gain	1 (4)
	SUFU	Copy number loss	1 (4)
	FOXA1	Copy number loss	1 (4)
	EED	Copy number loss	1 (4)
	MTAP	Copy number loss	1 (4)
	NF1	Copy number loss	1 (4)
	RASA1	Copy number loss	1 (4)
Rearrangements			

	NF1	SS18-NF1 fusion	1 (4)
	NAB2-STAT6	Chromosomal rearrangement	1 (4)
	DSCR4-ERG	Chromosomal rearrangement	1 (4)
